# How Mindfulness Affects Life Satisfaction: Based on the Mindfulness-to-Meaning Theory

**DOI:** 10.3389/fpsyg.2022.887940

**Published:** 2022-06-30

**Authors:** Xiaojun Li, Liping Ma, Qi Li

**Affiliations:** ^1^School of Teacher Education, Nanjing Xiaozhuang University, Nanjing, China; ^2^Department of Psychology, Hunan Normal University, Changsha, China; ^3^Institute of Early Childhood Education, Faculty of Education, Beijing Normal University, Beijing, China

**Keywords:** trait mindfulness, core self-evaluation, positive affect, negative affect, life satisfaction

## Abstract

Life satisfaction is the general evaluation of the individual’s life, which is of great significance to achieving a better life. The purpose of the present study was to investigate the mediating effect of core self-evaluation, positive affect, and negative affect in the relationship between trait mindfulness and life satisfaction based on the Mindfulness-to-Meaning theory. 991 Chinese undergraduates (692 females, 299 males) completed the Mindful Attention Awareness Scale, the Core Self-Evaluations Scale, the Positive Affect and Negative Affect Scale, and the Satisfaction with Life Scale. The results indicated that core self-evaluation and negative affect mediated the effect of trait mindfulness on life satisfaction, consistent with the Mindfulness-to-Meaning theory. Furthermore, trait mindfulness affected life satisfaction by the mediation paths of “core self-evaluation→positive affect” and “core self-evaluation→negative affect,” which uncovered the underlying mechanism of promoting life satisfaction by combining the point of view of cognition (core self-evaluation) and emotion (positive and negative affect). The present study not only contributes to a better theoretical understanding of how trait mindfulness links to life satisfaction but also provides valuable guidance for enhancing life satisfaction.

## Introduction

As a cognitive aspect of subjective well-being, life satisfaction is the general evaluation of the individual’s life, such as health conditions, social relations, and financial status (Röcke, 2021), which plays a prominent role in achieving a better life (Headey et al., 1993). Abundant studies have suggested that low life satisfaction is closely related to adverse health outcomes, such as depression, social anxiety, addictive behavior, substance abuse, and suicide (Koivumaa-Honkanen et al., 2001; Zullig et al., 2001; Koivumaa-Honkanen et al., 2004; Eng et al., 2005; Bellis et al., 2012; Rogowska et al., 2020). In utilitarian moral philosophy, life satisfaction is recognized as the ultimate goal of life (Ehrhardt and Veenhoven, 2000). Therefore, more research is needed to explore the underlying mechanism of improving life satisfaction.

### Mindfulness and Life Satisfaction

Previous studies have indicated that many internal-control factors make an impact on life satisfaction, like gratitude, resilience, self-control, and self-regulation (Kandemir, 2014; Bajaj and Pande, 2016; Li et al., 2016; Liang et al., 2022). Meanwhile, as a core variable of internal-control factors, mindfulness has been growing documented in the correlation with life satisfaction (Schutte and Malouff, 2011; Wang and Kong, 2014; Bajaj and Pande, 2016; Tan et al., 2016; Chen et al., 2017; Liang et al., 2022). Mindfulness is defined as the attention and awareness of the current experiences, characterized by non-critical observation and experience of current experience (Brown and Ryan, 2003; Brown et al., 2007). Generally speaking, the present studies can be divided into experimental and observational methods: for one thing, mindfulness based on intervention had a noteworthy impact on life satisfaction (Harnett et al., 2010; Henriksson et al., 2016; Lötzke et al., 2016; Chandrasekara, 2018; Amundsen et al., 2020; Gupta and Verma, 2020); for another thing, individuals with trait mindfulness tended to have higher scores of life satisfaction. Moreover, existing research has preliminarily expanded the path between mindfulness and life satisfaction. For example, emotional intelligence, self-control, and resilience mediated the relationship between mindfulness and life satisfaction (Schutte and Malouff, 2011; Bajaj and Pande, 2016; Tan et al., 2016; Chen et al., 2017; Liang et al., 2022). However, the underlying mechanism which combines cognition and emotion between mindfulness and life satisfaction is still unclear. Therefore, based on the Mindfulness-to-meaning theory, the present study aimed at exploring the underlying mechanism combining emotion and cognition between mindfulness and life satisfaction.

### Mindfulness, Core Self-Evaluation, and Life Satisfaction

The Mindfulness-to-Meaning theory proposed by Garland et al. (2015b) clarified that mindfulness generated well-being through the processes of attention, appraisal, and emotion. In particular, the theory suggested that mindfulness engendered well-being by promoting cognitive reappraisal, during which the individual rebuilt his or her ego and value (Garland et al., 2015a). On the one hand, life satisfaction has been widely recognized as the key indicator of well-being (Diener et al., 2018). On the other hand, core self-evaluation is an essential evaluation of the ability and value held by the individual (Judge et al., 1997). Therefore, based on the Mindfulness-to-Meaning theory, we speculated that core self-evaluation played a mediation role in the relationship between mindfulness and life satisfaction. Specifically, individuals with trait mindfulness were more likely to accept themselves in a non-critical way and had higher scores on core self-evaluation. They tended to hold the idea that they were able to control their lives, upon which life satisfaction could be promoted. Moreover, previous studies have demonstrated that core self-evaluation can partially mediate the impact of trait mindfulness on life satisfaction (Kong et al., 2014; Tan et al., 2016), which lays a foundation for the hypothesis of the present study.

### Mindfulness, Core Self-Evaluation, Positive Affect, Negative Affect, and Life Satisfaction

Meanwhile, according to the Mindfulness-to-Meaning theory, mindfulness contributes to positive cognitive-affective processing through cognitive reappraisal, which in turn enhances individuals’ well-being (Garland et al., 2015a). Taking this theory further, mindfulness reshapes the way that individuals typically focus on their experiences, so that they engage in positive cognitive reappraisals of themselves. For example, people will reconsider their failure as a way to galvanize their growth. Additionally, in the process of cognitive reappraisal, mindfulness would further strengthen the individual’s ability to regulate negative experiences and appreciate positive experiences, thereby facilitating life satisfaction (Garland et al., 2015b). That is to say, positive affect and negative affect might play a mediation role between not only mindfulness and life satisfaction but core self-evaluation and life satisfaction. Previous studies have provided preliminary evidence. On the one hand, behavioral research has provided empirical evidence for the interaction of cognition and emotion. Dennis (2010) proposed the Emotion-Cognition Integration model in a review of neuroscience research on emotion regulation, which provided neuroscientific support for the positive effect of emotion-cognition interaction on emotion regulation. Raschle et al. (2017) adopted the Affective Stroop task to explore the interaction of emotion and cognition, and fMRI results confirmed the neural correlation of the interaction between emotion and cognition. An ERP study based on mindfulness-based music training also showed that the alleviation of mindfulness on negative affect was closely linked to the interaction of emotion and cognition (Liu et al., 2021). On the other hand, several observational studies have revealed the correlation between mindfulness, core self-evaluation, positive affect, and negative affect. For one thing, mindfulness could effectively reduce negative affect in social interaction (Mandal et al., 2012; May et al., 2020), and promote positive affect (Malinowski and Lim, 2015). Besides, core self-evaluation was positively linked to positive affect and negatively linked to negative affect (Sudha and Shahnawaz, 2013). For another thing, Singh and Jha (2008) found that positive affect and negative affect were highly correlated with life satisfaction, and further studies demonstrated that positive affect had a positive impact on life satisfaction, while negative affect had the opposite effect (He et al., 2014; Extremera and Rey, 2016). As a result, positive affect and negative affect might serve as a mediator between trait mindfulness and life satisfaction, core self-evaluation, and life satisfaction as well.

### The Present Study

In summary, based on the Mindfulness-to-Meaning theory, this study aimed to explore the mediating role of core self-evaluation and positive and negative affect between mindfulness and life satisfaction, which contributes to our understanding of the impact of mindfulness on life satisfaction from the perspective of cognition and emotion. Accordingly, we proposed three hypotheses following: (1) core self-evaluation could mediate the path of trait mindfulness on life satisfaction. (2) positive affect and negative affect could mediate the path of trait mindfulness on life satisfaction. (3) trait mindfulness could influence life satisfaction by the mediation chain of “core self-evaluation→negative affect” and “core self-evaluation→positive affect.”

## Materials and Methods

### Participants and Procedure

Totally 991 undergraduate students (692 females and 299 males) were randomly recruited in cluster sampling. The participants were from several universities in China, aging from 17 to 26 (*M* = 19.05, SD = 1.54). Before the test, the research assistants made an explanation to the participants concerning the purpose and confidentiality of the survey. All the participant was told that they had the right to reject the questions that made them uncomfortable, and the freedom to withdraw from the survey at any time. After completing these questionnaires, we tried our best to guarantee the authenticity and confidentiality of their responses. Besides, all the participants who completed these questionnaires were handed out 10 RMB as compensation. The study has been approved by the ethical committee of the author’s organization. The above data were partly derived from the ongoing project “Early Adverse Environment Influences Cognitive Affective Mechanism”. Some data have been used in previous studies (Xiang and Yuan, 2021).

### Measure

#### Mindful Attention Awareness Scale

Mindful Attention Awareness Scale (MAAS) developed by Brown and Ryan (2003) and consisting of 15 items (e.g., “I prefer to walk fast to the destination rather than pay attention to the experience what happens on the road”) was used in the research. The scale is scored by a 6-point Likert scale (from 1 “almost always” to 6 “almost never”). Higher scores indicate higher mindfulness. In the present study, we employed an adaption used by Xiang et al. (2019), which was demonstrated high reliability and validity in Chinese groups. In this study, Cronbach’s alpha was 0.80.

#### The Satisfaction With Life Scale

The Satisfaction with Life Scale (SWLS, Diener et al., 1985) consisting of 5 items (e.g., “In most ways my life is close to my ideal”) was scored by a 7-point Likert scale (from 1 “strongly disagree” to 7 “strongly agree”) in the study. The higher the score indicates the better people are satisfied with their life. We employed the Chinese version of SWLS, which was proved to have high reliability and validity (Song et al., 2012; Kong et al., 2019; Xiang et al., 2020). In this study, Cronbach’s alpha was 0.84.

#### Positive Affect and Negative Affect Scale

The positive affect and negative affect Scale (PANAS) was developed by Watson et al. (1988), which consists of 10 items measuring positive affect (e.g., excited) and 10 items for negative affect (e.g., nervous). The PANAS is a 5-point scale (from 1 “very slightly”/“not at all” to 6 “extremely”). We used the Chinese version of this scale (Zhang et al., 2004) which has demonstrated good reliability and validity in Chinese groups (Xiang et al., 2020). The Cronbach’s alpha coefficients were 0.93 and 0.85 respectively.

#### The Core Self-Evaluations Scale

The Core Self-Evaluations Scale (CSES) was developed by Judge et al. (2003), which consisted of 10 items and was scored on a 5-point Likert-type scale. The higher scores mean higher self-evaluation. In this study, the adaption by Song et al. (2012) was used for our participants, which has shown good reliability and validity in Chinese groups (Xiang et al., 2019). In this study, the Cronbach’s alpha was 0.88.

### Statistical Analysis

SPSS 23.0 and AMOS 23.0 were used to conduct statistical analysis. Firstly, we used SPSS 23.0 to analyze the distribution of variables and the correlation between variables. Then, AMOS 23.0 was used to test the mediating effect of the hypothesis and the stability of the model. We took two steps to analyze the mediation effect. First, a measurement model was constructed in AMOS 23.0 to detect whether each potential variable could be well represented by its indicators. Three item parcels for mindfulness, positive affect, negative affect, and two for life satisfaction were created to control for inflated measurement errors (Little et al., 2002). Secondly, if the fitting degree of the measurement model was good, we continued to construct a structural model on this basis. For this reason, the chi-square statistic, standardized root-mean-square residual (SRMR, 0.080 or less), root-mean-square error of approximation (RMSEA, 0.080 or less), and comparative fit index (CFI, 0.900 or more) were used as an indicator of model fitting degree (Byrne, 2001). At the same time, Akaike Information Criterion (AIC) was used as the index to compare the model (Akaike, 1987) and expected cross-validation index (ECVI) to evaluate the replication potential of the model. Subsequently, we used 95% bias-corrected bootstrap method to evaluate the mediating effect significance (Fritz et al., 2012). Finally, multi-group confirmatory factor analysis was used to test the model’s transgender stability.

## Results

### Descriptive Statistics and Measurement Models

A total of 5 latent variables (mindfulness, core self-evaluation, life satisfaction, positive affect, and negative affect) and 14 observational variables were included in the measurement model. The results showed that the measurement data fit the model well [χ^2^ (67, 991) = 211.109, *p* < 0.001, RMSEA = 0.047, SRMR = 0.034, CFI = 0.981], indicating that all the potential variables were well represented by the observed variables. Table 1 shows that all latent variables in the measurement model are significantly correlated.

**TABLE 1:** Descriptive statistics and correlation analysis.

	M	SD	1	2	3	4	5
1. MF	60.91	9.08	1.000				
2. CSE	34.08	6.13	0.347***	1.000			
3. PA	29.76	7.61	0.080*	0.426***	1.000		
4. NA	18.73	5.74	−0.302***	−0.426***	0.086**	1.000	
5. LS	19.64	5.90	0.171***	0.483***	0.358***	−0.211***	1.000

*MF, mindfulness; CSE, core self-evaluations; PA, positive affect; NA, negative affect; LS, life satisfaction.*

***p < 0.001, **p < 0.01, *p < 0.05.

### Common Method Variance

Because of the questionnaire method, common method variance might exist in this study. Firstly, through Harman single factor test (Podsakoff et al., 2003, 2012), the variance interpretation percentage of the first common factor was 20.227%, less than 40%. Besides, the confirmatory factor analysis was used to examine the common method variance (Iverson and Maguire, 2000; Podsakoff et al., 2003). All variables were incorporated into one latent variable. The result indicated the fitting index is less than satisfactory [χ^2^/df = 12.988, CFI = 0.360, TLI = 0.335, NFI = 0.343, RMSEA = 0.110, SRMR = 0.145]. Therefore, there is no common method variance in this study.

### Structural Model

**FIGURE 1:**
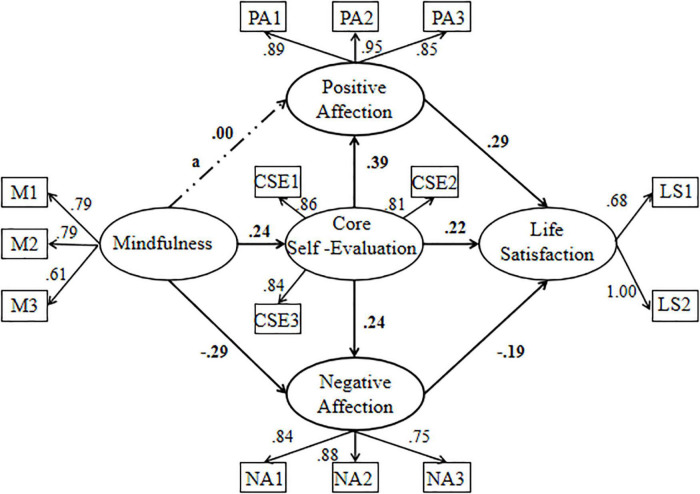
The chain mediation model (*N* = 991). M1, M2 and M3 are three parcels of mindfulness. LS1 and LS2 are two parcels of life satisfaction. PA1, PA2 and PA3 are three parcels of positive affect. CSE1, CSE2 and CSE3 are three parcels of core self-evaluation. NA1, NA2 and NA are three parcels of negative affect.

The results of regression analysis showed that mindfulness directly and significantly affected life satisfaction when other variables were missing (β = 0.171, *p* < 0.001). On this basis, we built a structural equation model, which included three mediating paths (core self-evaluation, positive affect, and negative affect) and two chain mediating paths (core self-evaluation→positive affect, core self-evaluation→negative affect). The results showed that the fit degree of model 1 was good [χ^2^ (69, 991) = 234.264, *p* < 0.001, RMSEA = 0.049, SRMR = 0.050, CFI = 0.978] (see Table 2), but the path coefficient between mindfulness and positive affect was not significant (β = 0.001, *p* = 0.969). Therefore, we built model 2 by limiting non-significant paths to zero in this Model. The results indicated that there was no significant difference between the two groups [χ^2^ (70, 991) = 234.266, *p* < 0.001, RMSEA = 0.049, SRMR = 0.050, CFI = 0.978]. However, according to the principle of model simplification, model 2 was more suitable, so we chose model 2 as the final Model (see Figure 1).

**TABLE 2:** Fit indices of model 1 and model 2.

	*X* ^2^	*df*	CFI	RMSEA	SRMR	AIC	ECVI
Model 1	234.69	96	0.978	0.049	0.0497	306.264	0.309
Model 2	234.266	70	0.978	0.049	0.0497	304.266	0.307

*RMSEA, root-mean-square error of approximation; SRMR, standardized root-mean-square residual; CFI, comparative fit index; AIC, Akaike information criterion; ECVI, expected cross-validation index.*

In addition, since the structural equation model involved many limitations of data distribution (Shrout and Bolger, 2002), we further used the Bootstrap method to test the stability of the mediation variables in the structural equation model (5,000 Bootstrap samples were extracted from the original data). If the confidence interval of the estimated path coefficient does not include 0, it can be inferred that the mediating effect is significant (MacKinnon et al., 2002; Fritz and MacKinnon, 2007). The results showed that core self-evaluation [95% CI = (0.017, 0.050)] and negative affect [95% CI = (0.019, 0.049)] were significant mediators between mindfulness and life satisfaction. Moreover, the two chain paths of “core self-evaluation→positive affect” [95% CI = (0.010, 0.025)] and “core self-evaluation→negative affect” [95% CI = (0.004, 0.011)] also played a significant mediating role between them (see Table 3).

**TABLE 3:** Standardized indirect effects and 95% confidence intervals.

Pathways	Estimate	Lower	Upper
MF→PA→LS	0.000	–0.013	0.014
MF→CSE→LS	0.030	0.017	0.050
MF→NA→LS	0.031	0.019	0.049
MF→CSE→PA→LS	0.016	0.010	0.025
MF→CSE→NA→LS	0.006	0.004	0.011

*MF, mindfulness; CSE, core self-evaluations; PA, positive affect; NA, negative affect; LS, life satisfaction.*

### Gender Difference

In order to test the stability of our results, we conducted a multi-group confirmatory factor analysis on model 1. Firstly, we used SPSS 23.0 to test whether there were sex differences in the five latent variables. The results showed that there were no significant sex differences in mindfulness [*t*_(991)_ = 0.192, *p* = 0.848], positive affect [*t*_(991)_ = −0.919, *p* = 0.358], negative affect [*t*_(991)_ = 0.366, *p* = 0.737] and life satisfaction [*t*_(991)_ = −0.098, *p* = 0.922], but significant sex differences in core self-evaluation [*t*_(991)_ = 2.983, *p* = 0.003] and males scored higher than females.

Based on the gender differences we had found, as suggested by Byrne (2009), we established an unconstrained structural path model (allowing free path estimations) and a constrained structural path model (limiting the path coefficients of the two sexes to be equal). The results indicated that there were significant differences between the two models [χ^2^_(41,991)_ = 81.65, *p* < 0.01]. At the same time, the two models show good fitness (see Table 4).

**TABLE 4:** The comparison of unconstrained model between constrained model.

	*X* ^2^ */df*	CFI	RMSEA	SRMR	AIC	ECVI
Unconstrained model	2.197	0.976	0.035	0.0527	504.911	0.511
Constrained model	2.152	0.971	0.034	0.0531	504.639	0.510

*CFI, comparative fit index; RMSEA, root-mean-square error of approximation; SRMR, standardized root-mean-square residual; AIC, Akaike information criterion; ECVI, expected cross-validation index.*

In addition, since χ^2^ was significantly affected by the large sample size, in order to improve the accuracy of the results, we used Critical Ratios of Differences (CRD) as an indicator to further investigate the cross-sex stability of the model. According to the decision rules, when the absolute value of CRD is greater than 1.96, there is a significant difference between the two parameters (Arbuckle, 2003). The results showed that only the path of “core self-evaluation→life satisfaction” was significantly different between different gender (CRDCSE→LS = −2.679), among which, in the male sample, the direct effect of core self-evaluation on life satisfaction was very weak. Therefore, there was a difference in the cross-gender comparison of the model.

## Discussion

Based on the Mindfulness-to-Meaning theory, this study explored the internal relationship between trait mindfulness and life satisfaction from the perspectives of cognition (core self-evaluation) and emotion (positive affect and negative affect). The results indicated that core self-evaluation and positive and negative affect were the mediators in the relationship. Furthermore, we found that “core self-evaluation→positive affect” and “core self-evaluation→negative affect” were two mediating chain paths between trait mindfulness and life satisfaction.

Firstly, the results confirmed hypothesis 1 that trait mindfulness could influence life satisfaction through core self-evaluation, corresponding with the existing studies (Kong et al., 2014; Tan et al., 2016) and the Mindfulness-to-Meaning theory (Garland et al., 2015a,b). Individuals with a high level of mindfulness are more willing to actively accept themselves and the events that happen to them, so they are more prone to accepting things that they cannot change, such as appearance and thoughts, than individuals with low mindfulness (Brown and Ryan, 2003; Carson and Langer, 2006), and the higher the degree of self-acceptance of individuals, with more possibilities they are to form a positive self-evaluation. In other words, individuals with a higher level of trait mindfulness have a higher core self-evaluation. Simultaneously, previous studies have also found that core self-evaluation is a predictor of life satisfaction (Hsieh and Huang, 2017; Gurbuz et al., 2018), whose mechanism lies in that individuals with a high core self-evaluation are convinced that they are fully capable of controlling their own lives and thus have a higher degree of satisfaction with life. Interestingly, we found there were gender differences in the relationship between core self-evaluation and life satisfaction. Based on the difference of thoughts between males and females, we attribute this difference to the men’s preference to promoting life satisfaction from emotion rather than core self-evaluation.

In addition, the results showed that only negative affect mediated between trait mindfulness and life satisfaction, while positive affect had no significant mediating effect, which was partially consistent with hypothesis 2. For one thing, mindfulness was defined as the adoption of a non-critical and an accepting attitude toward negative emotion, which promoted individuals to reduce emotional fluctuations (Brown and Ryan, 2003; Brown et al., 2007). According to the Mindfulness-to-Meaning theory, mindfulness is mainly adopted to inhibit negative affect toward stressful situations (Garland et al., 2015a). Mandal et al. (2012) have pointed out that mindfulness, to a large extent, actually affects individuals’ mental health by regulating their emotional balance, i.e., thus reducing negative affect rather than enhancing positive affect. Therefore, mindfulness has a more prominent effect on negative affect than positive affect. For another thing, the impact of negative experiences are stronger and longer than positive ones (Baumeister et al., 2001). That is to say, individuals who are exposed to negative affect may be easily regulated by mindfulness than positive affect. However, there are various conclusions in terms of the mediating role of positive affect in the current research. Some studies found that trait mindfulness could increase the positive affect and reduce the negative affect (Bajaj and Pande, 2016). Other studies concluded that, after 8-week mindfulness intervention training, the participants were found to better get rid of unpleasant experiences so as to maintain a positive experience (Schroevers and Brandsma, 2010). Therefore, the mediating role of positive affect between mindfulness and life satisfaction is needed to be further investigated.

More importantly, we found that trait mindfulness could affect life satisfaction through two chain mediations: “core self-evaluation→positive affect” and “core self-evaluation→negative affect.” This result supports hypothesis 3 and totally corresponds with the Mindfulness-to-Meaning theory. Based on this theoretical model, we speculated that it was precisely by influencing an individual’s cognition (core self-evaluation) that mindfulness influenced an individual’s emotion (positive and negative affect) and ultimately increased life satisfaction. Specifically, the internal mechanism may be that mindfulness can change the cognition of oneself by improving self-acceptance so as to hold more positive core self-evaluation (Kong et al., 2014; Liu et al., 2019). Moreover, core self-evaluation helps individuals to build self-confidence and endows themselves and external things with a positive attitude so that they experience more positive affect and less negative affect (He et al., 2014). At the same time, the more positive the individual’s emotion is, the easier to focus on the positive side of daily life and feel satisfied with it, and finally life satisfaction can be improved (Extremera and Rey, 2016).

In summary, this study novelly revealed the underlying mechanism between trait mindfulness and life satisfaction from the perspective of the Mindfulness-to-Meaning theory. The results of the study preliminarily found that both cognition and emotion are the important mediators that affect life satisfaction from trait mindfulness. Specifically, we concluded that trait mindfulness affects life satisfaction by the chain mediation mechanism of “core self-evaluation→positive affect” and “core self-evaluation→negative affect.” This research not only helps to expound the intrinsic mechanism of the effect of trait mindfulness on life satisfaction but also arouses important implications for improving an individual’s quality of life and promoting positive affect. In addition, this conclusion also confirmed that cognition and emotion were not two independent parts. As a manifestation of cognition, we concluded that core self-evaluation had an impact on life satisfaction through positive affect and negative affect. Future research can be expanded from the influence of emotion on cognition to comprehensively reveal the cognition-emotion interaction of mindfulness on life satisfaction.

However, this study also has several limitations inevitably. First, the study adopted self-reported method to explore the conclusion. Although showing a good reliability and validity of the measurement, there may exist social desirability bias so that alternative methods are needed to expand the topic in the future. Besides, all participants were Chinese undergraduates. The future research can take cultural, ethnic, socioeconomic status, age, gender identity and other diversity factors into account through large samples to improve the external validity of the conclusions. Finally, the present study uncovered the underlying mechanism between trait mindfulness and life satisfaction through cognition and emotion, which corresponded with the Mindfulness-to-Meaning theory to some extent. Meanwhile, in addition to life satisfaction, the Mindfulness-to-Meaning theory also demonstrated that mindfulness could further generate a sense of meaning through well-being. Future research is needed to test whether mindfulness can not only promote life satisfaction but also generate sense of meaning through the cognitive-affective processes, which totally verifies the Mindfulness-to-Meaning theory.

## Data Availability Statement

The datasets supporting the conclusions of the present study are not publicly available due to privacy concerns however they will be made available upon reasonable request from the corresponding authors.

## Ethics Statement

The studies involving human participants were reviewed and approved by the Academic Committee of the School of Psychology of Hunan Normal University. Written informed consent to participate in this study was provided by the participants’ or their legal guardian/next of kin.

## Author Contributions

XL made a contribution to the design of this manuscript, data analysis, and manuscript revising. LM made a contribution to conceiving the framework of theory, data analysis, writing, and revising the manuscript. QL made a contribution to manuscript writing and data analysis. All authors contributed to the article and approved the submitted version.

## Conflict of Interest

The authors declare that the research was conducted in the absence of any commercial or financial relationships that could be construed as a potential conflict of interest.

## Publisher’s Note

All claims expressed in this article are solely those of the authors and do not necessarily represent those of their affiliated organizations, or those of the publisher, the editors and the reviewers. Any product that may be evaluated in this article, or claim that may be made by its manufacturer, is not guaranteed or endorsed by the publisher.
