# Corrigendum: How mindfulness affects life satisfaction: Based on the mindfulness-to-meaning theory

**DOI:** 10.3389/fpsyg.2022.1056856

**Published:** 2022-10-13

**Authors:** Xiaojun Li, Liping Ma, Qi Li

**Affiliations:** ^1^School of Teacher Education, Nanjing Xiaozhuang University, Nanjing, China; ^2^Department of Psychology, Hunan Normal University, Changsha, China; ^3^Institute of Early Childhood Education, Faculty of Education, Beijing Normal University, Beijing, China

**Keywords:** trait mindfulness, core self-evaluation, positive affect, negative affect, life satisfaction

In the published article, there was an error in affiliation 3. Instead of Department of Psychology, Beijing Normal University, Beijing, China, it should be Institute of Early Childhood Education, Faculty of Education, Beijing Normal University, Beijing, China.

The authors apologize for this error and state that this does not change the scientific conclusions of the article in any way. The original article has been updated.

## Publisher's note

All claims expressed in this article are solely those of the authors and do not necessarily represent those of their affiliated organizations, or those of the publisher, the editors and the reviewers. Any product that may be evaluated in this article, or claim that may be made by its manufacturer, is not guaranteed or endorsed by the publisher.

